# Acute Kidney Injury in Non-Intensive Care Unit (ICU) Hospitalizations for Coronavirus Disease (COVID-19)

**DOI:** 10.3390/pathogens11111272

**Published:** 2022-10-31

**Authors:** Fabrizio Fabrizi, Carlo M. Alfieri, Paolo Molinari, Francesco Tamborini, Marianna Tangredi, Anna Sikharulidze, Francesco Blasi, Anna Fracanzani, Walter Monzani, Flora Peyvandi, Giuseppe Castellano

**Affiliations:** 1 Division of Nephrology, Dialysis and Kidney Transplant, Foundation IRCCS Cà Granda Ospedale Maggiore Policlinico, 20122 Milano, Italy; 2 Department of Clinical Sciences and Community Health, University School of Medicine, 20122 Milano, Italy; 3 Respiratory Unit and Adult Cystic Fibrosis Center, Foundation IRCCS Cà Granda Ospedale Maggiore Policlinico, 20122 Milan, Italy; 4 Department of Pathophysiology and Transplantation, University School of Medicine, 20122 Milano, Italy; 5 Division of Metabolic Internal Medicine, Foundation IRCCS Cà Granda Ospedale Maggiore Policlinico, 20122 Milano, Italy; 6 Division of Sub-Intensive Care Medicine, Foundation IRCCS Cà Granda Ospedale Maggiore Policlinico, 20122 Milano, Italy; 7 Division of Internal Medicine, Angelo Bianchi Bonomi Hemophilia and Thrombosis Center, Foundation IRCCS Cà Granda Ospedale Maggiore Policlinico, 20122 Milano, Italy

**Keywords:** acute kidney injury, chronic kidney disease, COVID-19, death, SARS-CoV-2

## Abstract

Background: Acute kidney injury (AKI) is a common complication among SARS-CoV-2-positive patients who undergo hospitalization. Abundant evidence exists concerning the epidemiology of AKI in patients hospitalized in the ICU for COVID-19 but limited data are available about the occurrence of AKI in SARS-CoV-2-positive patients being hospitalized in a non-ICU setting. Aim and Methods: We have carried out a retrospective study to evaluate frequency and risk factors for AKI among patients consecutively admitted at a third-level university hospital starting from February 2020 (the beginning of the first wave of the SARS-CoV-2 pandemic); all patients were hospitalized outside the ICU. Results: A total of 387 SARS-CoV-2-positive patients were included in the current study; 372 (96.1%) had SARS-CoV-2-related pneumonia. In-hospital AKI onset was recorded in 119 (30.7%) patients, mainly with AKI stage 1 (*n* = 74, 62.2%); eighteen (4.6%) patients reported AKI stage 3 and six (1.5%) patients had HD-dependent AKI. There were 235 (60.7%) patients with severe COVID-19, and this was more common in patients developing AKI, 94.5% (86/119) vs. 86.1% (149/268), *p =* 0.02. Multivariate regression model (*n* = 144 patients) reported an independent and significant relationship between AKI occurrence and greater levels of ferritin (*p =* 0.036), IL-6 (*p =* 0.032), and azotemia at admission (*p* = 0.0001). A total of 69 (17.8%) SARS-CoV-2-positive patients died and strong predictors of in-hospital death resulted from age (*p* < 0.0001), serum ferritin (*p* < 0.0001) and white blood cells (*p* < 0.001). According to multivariable analysis (*n* = 163 patients), there was a consistent link between in-hospital death and AKI stage (1) (*p* = 0.021) and -stage (2) (*p* = 0.009). Our results support the notion that AKI occurs frequently among hospitalized COVID-19 patients even in a non-ICU setting and plays a pivotal role in the mortality of this population. Further studies are ongoing in order to clearly establish the frequency of AKI in patients with COVID-19; the mechanisms underlying kidney injury in this population are an area of active investigation. These data provide solid evidence to support close monitoring of COVID-19 patients for the development of AKI and measures taken to prevent this.

## 1. Introduction

Coronavirus disease 2019 (COVID-19) is caused by the novel Severe Acute Respiratory Syndrome Coronavirus 2 (SARS-CoV-2); it was originally reported in Wuhan, China and declared a global pandemic on 11 March 2020 [1]. An update from the World Health Organization (2 August 2022) reports that globally the number of confirmed cases of SARS-CoV-2 is 575,887,049 including 6,398,412 deaths, reported to WHO [2].

The first epidemic cluster of COVID-19 recorded in Milan was in February 2020; in the weeks that followed, Milan became one of the most important epicentres for COVID-19. Solid evidence has been rapidly accumulated showing that SARS-CoV-2 infection has various clinical manifestations; COVID-19 patients can be asymptomatic and at least a third of people who are SARS-CoV-2-infected do not have noticeable symptoms. Alternatively, patients with COVID-19 can develop mild abnormalities of the upper respiratory tract, or show viral pneumonia and eventually respiratory failure.

Although many patients with COVID-19 manifest respiratory tract symptoms, SARS-CoV-2 infection can target several organs/systems including the kidneys [3]. Acute kidney injury (AKI) is now considered an important complication of SARS-CoV-2 infection; according to a recent systematic review of the literature with meta-analysis of clinical studies (*n* = 39 reports, *n* = 25,566 unique patients), the pooled incidence of AKI was 15.4% (95% CI, 0.107; 0.21, *p* < 0.0001) across the studies [4]. The frequency of AKI was much greater among patients with severe COVID-19 (50%, range, 42–63%), and such remarkable heterogeneity is probably related to numerous factors including patient characteristics, illness severity, or differences in everyday clinical activities [4].

Numerous surveys have evaluated the frequency and risk factors for AKI in patients with SARS-CoV-2 infection; it appears that the information in the medical literature regarding the occurrence of AKI in patients referred to non-intensive medical departments is much lower compared with that described in patients admitted to intensive care [5]. The aim of this study is to report on incidence, risk factors, and outcomes of AKI in a population of COVID-19 patients who underwent hospitalization in a non-intensive care unit (ICU) setting at a metropolitan hospital in Milan. Milan is one of the cities most affected by the SARS-CoV-2 pandemic in the world.

## 2. Methods

### 2.1. Study Design and Ethics

**Figure 1:**
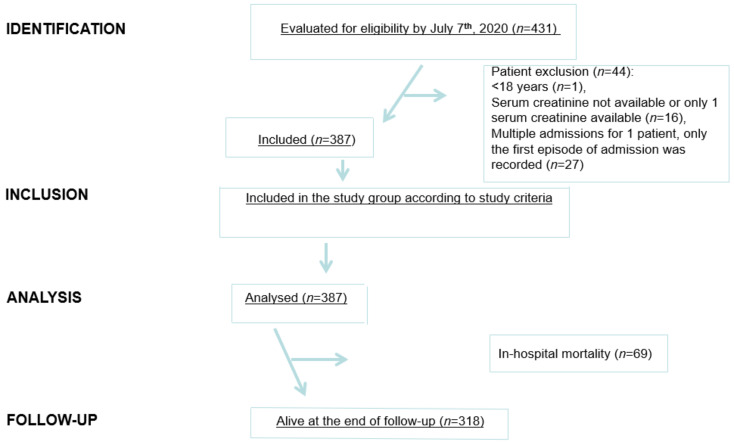
STROBE flow chart. STROBE, STrengthening the Reporting of OBservational Studies in Epidemiology.

This was an investigator-initiated, single-centre, retrospective study. Data came from the electronic health records from Maggiore Policlinico Hospital and IRCCS Ca’ Granda Foundation located in Milan midtown. The research involved analysis of anonymised data routinely collected in the course of standard care and written informed consent was waived due to the retrospective design of the study and pandemic course of the disease. Data were analysed and interpreted by the authors who reviewed the manuscript and confirmed the accuracy and completeness of the data and adherence to the protocol. The study was conducted in compliance with the Declaration of Helsinki, and International Council for Harmonization Guidelines for Good Clinical Practice. The results have been shown according to the STROBE guidelines [6] (Figure 1).

### 2.2. Study Population and Inclusion Criteria

Maggiore Policlinico Hospital serves a large racially and ethnically diverse patient population. During the surge of COVID-19 in the Milan area (2020), almost all elective admissions were cancelled. It should be noted that the first confirmed case of SARS-CoV-2 infection at Maggiore Policlinico Hospital was recorded on 23 February 2020.

We included patients (at least 18 years old) who were consecutively admitted to the Maggiore Policlinico Hospital from 22 February until 27 July 2020. All individuals enrolled in the study needed hospital admission due to clinical or radiological evidence of pneumonia or acute respiratory distress or influenza-like illness. All patients underwent pharyngeal swabbing and SARS-CoV-2 infection was detected by reverse transcriptase–polymerase chain reaction (RT-PCR) assay.

### 2.3. Exclusion Criteria

We excluded patients with known end-stage renal disease prior to admission and patients who were hospitalized for <48 h. Patients hospitalized in the ICU setting were excluded.

### 2.4. Data Collection and Measurements

Demographic status, comorbidities, medical therapies, vital signs, physical examination findings, laboratory findings, and chest X-ray findings were recorded from all patients. Demographics included age, gender, race and ethnicity. Study data were obtained from the electronic health record, which is an integrated electronic health record including in-patient and out-patient visits in the health system. Laboratory data consisted of complete blood count, haemostasis parameters, kidney and liver function, high-sensitivity C-reactive protein (PCR), and serum cytokines. Range values of biochemistries were as follows: serum creatinine, 0.7–1.2 mg/dL; azotemia, 15–50 mg/dL; PCR, <0.5 mg/L; interleukin-6, 2–29 pg/mL; D-dimer, <500 mcg/mL; white blood cells, 4000–10,000/mL; lymphocytes, 1500–3000/mL; alanine aminotransferase (ALT), 9–50 IU/L; lactic dehydrogenase (LDH), 135–225 IU/L; ferritin, 30–400 ng/mL. Arterial hypertension was defined as an increase of systolic blood pressure over 140 mmHg, and a diastolic increase to more than 90 mmHg. Serial monitoring of these laboratory tests was carried out for each patient according to the patient’s clinical progress.

### 2.5. Definitions

The primary outcome was AKI; the Kidney Disease: Improving Global Outcome (KDIGO) definition was adopted to identify AKI [7]. AKI was defined using a 0.3 mg/dL increase or >50% increase in serum creatinine from the baseline creatinine to maximum in-hospital creatinine. Baseline creatinine was defined as the mean creatinine value between 7 and 365 days before hospitalization. In those without a baseline serum creatinine, the minimum creatinine value during hospitalization was adopted as the baseline creatinine as reported by Siew and colleagues [8]. AKI severity was staged according to Kidney Disease: Improving Global Outcome (KDIGO) criteria: stage (1), increase in serum creatinine by 0.3 mg/dL (26.5 mmol/L) or a 50–99% increase in serum creatinine; stage (2) 100–199% increase in serum creatinine; stage (3) 200% or more increase, or increase in serum creatinine to ≥4mg/dL (353.6 mmol/L) or initiation of renal replacement therapy. Those patients who required renal replacement therapy automatically met the definition of stage 3 AKI. Urine output criteria were not used to define AKI as urine output was not reliably collected. Patients with suspected AKI were reviewed on a case-by-case basis to confirm the diagnosis.

The definition of COVID-19 pneumonia was made on the basis of radiographic abnormalities in the lung. Severe COVID-19 pneumonia was defined as meeting any of the following conditions: (1) respiratory >30 breaths/min, (2) oxygen saturation <93% in a resting state, (3) PF ratio (arterial oxygen partial pressure [Pa0_2_]/fractional inspired oxygen [Fi0_2_]) <300 mmHg.

### 2.6. Outcomes

The primary outcome was in-hospital AKI, and the secondary outcome included in-hospital death. Patient data were censored at discharge from hospital, death, or transfer to other department or other hospitals, whichever occurred first.

### 2.7. Statistical Analyses

Data conforming to normal distribution were reported as mean (±standard deviation); alternatively, medians were adopted for data with non-normal distribution. Comparison of data was made by chi-squared test, *t* Test, and Wilcoxon rank test. Multivariate analysis (logistic regression model) was adopted to test the association of covariates of interest (including diagnosis of COVID-19) with the risk of development of AKI. The demographic, biochemical, and other parameters that were relevant from the clinical standpoint in our cohort of SARS-CoV-2-positive patients were included in the model. Specifically, variables with *p* < 0.05 in the univariate analysis entered into multivariate analysis to select predictors (inclusion criteria was *p* < 0.05 and exclusion criteria *p* < 0.10). The association between AKI and death was evaluated both as a dichotomous variable and as an ordinal categorical variable testing the contribution of each AKI stage as compared to subjects without AKI. Logistic regression models were also used to estimate the risk of in-hospital death for patients with AKI in comparison with patients without AKI in our cohort of SARS-CoV-2-positive patients. Results are presented as odds ratios (ORs) with 95% confidence intervals (95% CIs) and *p* values. All statistical analyses were performed using SPSS version 2.0 software (IBM Corp.).

## 3. Results

### 3.1. Baseline Characteristics

A total of 387 patients were included in the current study. All of them were SARS-CoV-2-positive, 372 (96.1%) had SARS-CoV-2-related pneumonia. The mean age was 65.4 ± 16.3 years (Table 1) and the frequency of comorbidities ranged between 9.8% and 44.9% (Table 1). The prevalence of arterial hypertension and diabetes was 44.9% (174/387) and 16.8% (65/387), respectively. Eight-six (22.2%) patients had renin-angiotensin system (RAS) blockade at admission. As listed in Table 2, 72 (18.6%) individuals underwent mechanical ventilation during their hospital stay.

### 3.2. Acute Kidney Injury

One hundred and nineteen (30.7%) patients developed AKI during their hospital stay (Table 2), 18 (4.6%) reported AKI stage 3 and six (1.5%) patients had HD-dependent AKI. There were significant differences between patients developing AKI or not at hospital admission (Table 3) and during their in-hospital stay (Table 4); patients developing AKI were older and had more frequent comorbidities (arterial hypertension, peripheral arterial disease, and chronic kidney disease) than patients who did not. There were 235 patients with severe COVID-19, and this was more common in patients developing AKI (*p* = 0.02). The unadjusted OR for severe COVID-19 was 1.68 (95% CI, 1.19–2.38, *p* < 0.003) among AKI-positive versus AKI-negative patients. The characteristics of severe COVID-19 positive study patients (hospital admission and in-hospital stay) have been listed in Supplementary Tables S1 and S2.

Multivariate regression analysis reported an independent and significant relationship between in-hospital AKI development and greater levels of ferritin (*p* = 0.036), azotemia (*p* = 0.0001), and IL-6 (*p* = 0.032) (Table 5, *n* = 144 patients).

### 3.3. Mortality

In this cohort study, 69 (17.8%) patients died during the observation period, out of 387 patients. Most deaths occurred in patients with critical illness. Univariate analysis reported significant differences between patients who survived and those who did not with regard to several parameters at hospital admission (Table 6 and Table 7) and during hospital stay (Table 8). Death occurred more frequently in those patients with severe COVID-19 (*p* = 0.04).

Multivariable analysis shows that there was an independent and significant relationship between death rate and greater levels of ferritin (*p* = 0.0001), and ageing (*p* = 0.0001), or occurrence of cardiomyopathy (*p* = 0.002) (Table 9; *n* = 284 patients). Another logistic regression model reported an independent and significant association between mortality and greater levels of white blood cells (*p* = 0.0001), and AKI stage (1) (*p* = 0.021) and -stage (2) (*p* = 0.009) (Table 10; *n* = 165 patients).

Using the group without AKI as the reference cohort, the unadjusted OR for in-hospital death was 3.85 (2.24; 6.60, *p* < 0.001). Stage 1 AKI played a pivotal role in increasing in-hospital death risk among patients positive for SARs-CoV-2 infection; AKI remained significant following adjustment for comorbid conditions (Table 11).

## 4. Discussion

We found a high frequency (30.7%) of patients developing AKI in our patients who were hospitalized in a non-intensive care unit for SARS-CoV-2 infection. Recent evidence suggests that AKI in COVID-19 populations occurs as a result of SARS-CoV-2-specific factors such as virus-mediated injury, abnormal inflammatory response (cytokine storm), angiotensin II pathway activation, microangiopathy and hypercoagulative condition [9]. COVID-19-mediated kidney injury remains unclear; experimental studies in human kidney proximal tubular epithelial cells have indicated persistent infection with SARS-CoV-2 [10]. Virus particles in cytoplasm of proximal/distal tubular cells and podocytes have been found in COVID-19 patients, by electronic microscopy [11,12]. These findings may explain the occurrence of proteinuria, haematuria, and collapsing glomerulopathy in patients with SARS-CoV-2 infection [4,13,14].

The findings coming from the current study are in keeping with these assumptions as high levels of serum ferritin, and IL-2 were recorded as risk factors for in-hospital AKI in our regression logistic models. These SARS-CoV-2-specific factors probably interact with other well-known risk factors for AKI conferring vulnerability to the kidneys (i.e., hypovolemic conditions, nephrotoxic medications, or contrast media) [3].

The frequency of AKI in our population was greater than that found in COVID-19 patients from other countries who underwent hospitalization in ICU units [4]. The variation in the incidence of AKI in COVID-19 patients from various countries or regions may be explained in part by variable inclusion criteria (intensive care and all hospital admissions). In addition, the high frequency of AKI observed in the current study can be related to the large prevalence of comorbid conditions (arterial hypertension, 44.9%; cardiomyopathy, 25.3%), and patient characteristics (mean age, 66 ± 15.1 years). As an example, COVID-19 patients from Asia were younger and had fewer comorbidities than that reported in the current study, despite being hospitalized in an ICU setting [4]. Thus, we feel that the frequency of AKI in COVID-19 positive patients does not appear to be strongly related to the hospitalization setting (ICU or not). Patients included in the current study were hospitalized at the Maggiore Policlinico Hospital during the first surge of SARS-CoV-2 infection and Milan was an important epicentre of the COVID-19 pandemic. It is possible that some patients underwent hospitalization in a non-ICU setting as the intensive care units were overcrowded at that time.

We noted a death rate of 17.8% in our cohort and this confirms data from other study groups (COVID-19 patients hospitalized in non-intensive medical departments, 11.6%) [5]. In studies of AKI in COVID-19 patients referred to ICU settings, the hospital mortality ranged from 21.2% to 40.6% in the US and 4.4% to 44.3% in China [15,16,17,18,19,20,21,22,23,24,25,26,27,28,29]. Our findings suggest that AKI is a strong and independent risk factor for mortality in patients with COVID-19, associated with a three-fold increase in the odds of in-hospital deaths. The impact of AKI on mortality in COVID-19 has been reported in only a few studies to date. Among 701 patients with COVID-19, AKI stages 2 and 3 were associated with increasing hazard ratios for in-hospital death (stage 2, HR 3.51, 95% CI, 1.49–8.26 and stage 3, HR 4.35, 95% CI (Confidence Interval), 2.31–8.31) [15]. The low number of people with AKI stage 3 precluded the study concerning the impact of AKI stage 3 on mortality in our population. On the other hand, it is difficult to assess the impact of AKI on the death rate according to SARS-CoV-2 infection status in the light of numerous comorbidities reported in the study cohort.

Previous studies have indicated that SARS-CoV-2 uses angiotensin converting enzyme 2 (ACE2) as a cell entry receptor, prompting some authors to suggest that treatment with ACEI or ARB may increase the risk of severe complications associated with COVID-19 [30]. This has been questioned by other investigators, and we noted no increase in mortality associated with ACEI or ARB use [31].

The retrospective and database nature of the study comes with some limitations. First, we have not been able to collect ad-hoc important variables such as urinary outputs, and advanced serum and urinary inflammatory markers that are not routinely analysed or recorded. As an example, no data on renal pathology and SARS-CoV-2 detection in urine samples were available to estimate the activity of SARS-CoV-2 infection at the kidney level, and biomarkers to make a difference between pre-renal AKI from ATN (Acute Tubular Necrosis) were not collected. We had no information on baseline creatinine measurement prior to hospitalization and we used the lowest in-hospital creatinine value as the baseline creatinine in the analysis as a proxy for pre-hospital creatinine. We acknowledge that relying on the lowest in-hospital creatinine value may have led to under-estimation of the AKI events, nonetheless the use of the lowest in-hospital creatinine has been proven to be appropriate and has been widely adopted in many surveys [8]. The current study relied on automated health record data extraction; information for goals of care discussion was not obtainable and we were unable to assess circumstances around the withholding of dialysis. Finally, we have not included in our retrospective survey a COVID-19 negative AKI control group; thus, the magnitude of unmeasured confounders cannot be detailed.

In conclusion, AKI is a common complication of SARS-CoV-2 infection even in medical departments (outside the ICU). The death rate was not negligible and various factors (i.e., age, serum ferritin, white blood cells, among others) were independent risk factors for mortality in a non-intensive care setting including AKI (even at stage 1). Further studies with longer observation periods are required to understand the long-term impact of COVID-19 on the kidneys.

## 5. Disclosures

All authors declare that they have no competing interests, no support from any organization for the submitted work, and no financial relationship with any organizations that might have an interest in the submitted work.

This study has been presented (poster presentation) in part at the Annual Meeting of the Italian Society of Nephrology (62th Congress, Rimini, 6–9 October 2021).

## Figures and Tables

**Table 1:** Characteristics of COVID-19 positive study patients (admission).

Patients, *n*	*n* = 387
Age, years	66.0 ± 15.81
Males, *n*	247/387 (63.8%)
Positive medical history:	
Arterial hypertension, *n*	174/387 (44.9%)
Cardiomiopathy, *n*	98/387 (25.3%)
Chronic kidney insufficiency, *n*	40/387 (10.3%)
Chronic obstructive pulmonary disease (COPD), *n*	38/387 (9.8%)
Diabetes mellitus, *n*	65/387 (16.8%)
Malignancy, *n*	53/387 (13.7%)
Peripheral arterial disease, *n*	50/387 (12.9%)
Biochemistry at admission:	
Serum creatinine, mg/dL	1.59 ± 7.34
Azotemia, mg/dL	51.9 ± 44
PCR, mg/L	11.85 ± 21.9
IL-6, pg/mL	91.9 ± 127.9
D-dimer, mcg/mL	4420.1 ± 22,368
White blood cells, *n*	8234.3 ± 4594.8
Lymphocytes, *n*	1250.9 ± 2154.9
ALT, IU/L	67.0 ± 216.7
LDH, IU/L	342.3 ± 177.3
Ferritin, ng/mL	876 (26,714; 69)
Physical parameters at admission:	
Blood pressure, mmHg	131.1 ± 19.1/75.8 ± 12.0
Heart rate, bpm	88.4 ± 17.3
Body temperature, degree	37.9 ± 7.8
PaO_2_/FiO_2_	267.12 ± 102.5
(Stage 0) PaO_2_/FiO_2_ (>300)	153 (46.7%)/328
(Stage 1) PaO_2_/FiO_2_ (300–201)	89 (27.1%)/328
(Stage 2) PaO_2_/FiO_2_ (200–101)	60 (18.3%)/328
(Stage 3) PaO_2_/FiO_2_ (≤100)	26 (7.9%)/328
Medical therapy at admission:	
Angiotensin Converting Enzymes ACEIs, *n*	47/386 (12.2%)
Angiotensin Receptor Blockers ARBs, *n*	39/386 (10.1%)

**Table 2:** Characteristics of COVID-19 positive study patients (in-hospital stay).

Patients, *n*	*n* = 387
AKI	119/387 (30.7%)
AKI stage 1	74/118 (62.7%)
AKI stage 2	26/118 (22%)
AKI stage 3	18/118 (15.2%)
Respiratory support:	
Low flow oxygen delivery, *n*	202/387 (52.2%)
High flow oxygen delivery, *n*	192/387 (49.6%)
Mechanical ventilation, *n*	72/387 (18.6%)
Clinical manifestations (and adverse events) during hospital stay:	
SARS-CoV-2 -related pneumonia, *n*	372/387 (96.1%)
Anaemia, *n*	11/387 (2.8%)
Atrial fibrillation, *n*	8/387 (2.0%)
Haemodialysis, *n*	6/387 (1.5%)
Ischemic stroke, *n*	2/387 (0.05%)
Multi-organ failure, *n*	10/387 (2.6%)
Pleural effusion, *n*	3/387 (0.07%)
Posterior reversible encephalopathy syndrome (PRES), *n*	2/387 (0.05%)
Sepsis, *n*	67/387 (17.3%)
Thrombosis, *n*	23/387 (5.9%)
Urinary tract infection (UTI), *n*	2/387 (0.05%)
Concurrent medical therapy:	
ACEIs and/or ARBs, *n*	140/386 (36.3%)
Antibiotics, *n*	319/386 (82.6%)
Antivirals, *n*	147/386 (82.6%)
Diuretics, *n*	133/386 (34.4%)
FANS, non-steroidal anti-inflammatory drugs, *n*	87/387 (22.4%)
Glucocorticoids, *n*	159/385 (41.3%)
Heparin, *n*	326/386 (84.4%)
Hydroxychloroquine, *n*	342/385 (88.8%)

**Table 3:** COVID-19 patients developing AKI or not: univariate analysis (admission).

Patients, *n*	AKI (*n* = 119)	No AKI (*n* = 268)	*p*
Age, years	73.0 ± 14.9	62.2 ± 15.8	0.0001
Males, *n*	79 (66.4%)	168 (62.7%)	NS
Systolic BP, mmHg	125.3 ± 21	132.3 ± 18	0.04
Diastolic BP, mmHg	69.7 ± 16.2	76.7 ± 11.3	0.001
Heart rate, bpm	86.9 ± 20	88.5 ± 15.3	NS
Positive medical history:			
Arterial hypertension, *n*	73 (61.3%)	101 (41%)	0.0001
Cardiomiopathy, *n*	39 (32.7%)	59 (22%)	0.02
Chronic kidney insufficiency, *n*	22 (18.5%)	18 (6.7%)	0.0001
COPD, *n*	12 (10%)	26 (9.7%)	NS
Diabetes mellitus, *n*	25 (21%)	40 (14.9%)	NS
Malignancy, *n*	20 (16.8%)	33 (12.3%)	NS
Peripheral arterial disease, *n*	27 (22.7%)	23 (8.6%)	0.0001
Biochemistries at admission:			
Creatinine, mg/dL	1.82 ± 0.98	1.49 ± 8.8	NS
Azotemia, mg/dL	80.1 ± 57.2	38.8 ± 28.1	0.001
Oxigen delivery at admission:			
Low flow (Nasal cannulas/masks)	64 (53.8%)	138 (51.5%)	NS
High flow (C-PAP/HFNC)	69 (57.9%)	123 (45.9%)	0.01
Mechanical ventilation	22 (18.5%)	50 (18.9%)	NS
Medical therapy at admission:			
ACEIs, *n*	22 (18.5%)	25 (9.4%)	0.01
ARBs, *n*	16 (13.4%)	23 (8.6%)	NS
At admission:			
PaO_2_/FiO_2_	237.0 ± 107.1	286.2 ± 100	0.001
Body temperature, degree	37.3 ± 4.7	37.9 ± 7.5	NS

**Table 4:** COVID-19 patients developing AKI or not: univariate analysis (admission and in-hospital stay).

Patients, *n*	AKI (*n* = 119)	No AKI (*n* = 268)	*p*
Medical therapy during hospital stay:			
Antibiotics, *n*	108 (91.5%)	211 (78.7%)	0.001
Antivirals, *n*	42 (35.3%)	105 (39.3%)	NS
Diuretics, *n*	49 (41.5%)	84 (31.3%)	0.035
FANS, *n*	28 (23.5%)	59 (22%)	NS
Glucocorticoids, *n*	59 (50%)	100 (37.4%)	0.01
Heparin, *n*	101 (85.5%)	225 (83.9%)	NS
Hydroxychloroquine, *n*	103 (87.3%)	239 (89.5%)	NS
ACEIs/ARBs, *n*	48 (40.3%)	92 (34.3%)	NS
Clinical parameters during in-hospital stay:			
Multi Organ Failure (MOF), *n*	7 (5.9%)	3 (1.1%)	0.01
Haemodialysis (HD), *n*	6 (5%)	0	0.001
Severe COVID-19, *n*	86 (94.5%)	149 (86.1%)	0.02
COVID-19 related pneumonia, *n*	117 (98%)	255 (95.1%)	NS
Sepsis, *n*	31 (26%)	36 (13.4%)	0.002
Biochemistries at admission:			
IL-6, pg/mL	159.6 ± 195	65.3 ± 74	0.0001
LDH, IU/L	399.8 ± 197.8	316.78 ± 161.4	0.001
PCR, mg/dL	12.2 ± 10	11.6 ± 25.5	NS
D-dimer, mcg/mL	5663.64 ± 26791.1	3873.6 ± 20173.3	NS
Ferritin, ng/mL	2264.3 ± 3518,4	1103.1 ± 1145.7	0.001
ALT, IU/L	99.3 ± 375.5	53.1 ± 78.9	NS

**Table 5:** Multivariable logistic regression to identify risk factors for AKI in COVID-19 positive patients (*n* = 144 pts).

	B	SE	Wald Test	*p*	Exp (B)
LDH	0.001	0.001	0.872	0.350	1.001
IL-6	0.006	0.003	4.578	0.032	1.006
Ferritin	0.000	0.000	4.384	0.036	1.000
Azotemia	0.039	0.010	15.651	0.000	1.040
Arterial hypertension	0.087	0.532	0.027	0.871	1.090
Peripheral arterial disease	0.534	0.734	0.529	0.467	1.706
Cardiomiopathy	0.110	0.610	0.032	0.857	1.116
COPD	−3.77	1.949	3.754	0.053	0.023
ACEIs/ARBs	−0.366	0.509	0.516	0.472	0.694
Constant	−4.528	0.854	28.107	0.0001	0.011

**Table 6:** Exitus in COVID-19 positive patients: univariate analysis (admission).

Patients, *n*	Exitus (*n* = 69)	No Exitus (*n* = 318)	*p*
Age, years	78 ± 12	63 ± 15	0.001
Males, *n*	44/69 (63.8%)	203/318 (63.8%)	NS
Body temperature, degree	37.3 ± 1.08	37.9 ± 7.9	NS
Positive medical history:			
Arterial hypertension, *n*	47/69 (68%)	127/318 (39.9%)	0.001
Cardiomiopathy, *n*	36/69 (52.1%)	62/318 (19.5%)	0.001
Chronic kidney insufficiency, *n*	15/69 (21.7%)	25/318 (7.9%)	0.02
COPD, *n*	14/69 (20.2%)	24/318 (7.5%)	0.001
Diabetes mellitus, *n*	16/69 (23.1%)	49/318 (15.4%)	NS
Malignancy, *n*	21/69 (30.4%)	32/317 (10%)	0.001
Peripheral arterial disease, *n*	24/69 (34.8%)	26/318 (8.2%)	0.001
Biochemistries at admission:			
Creatinine, mg/dL	1.44 ± 0.6	1.62 ± 8.1	0.001
Azotemia, mg/dL	75.0 ± 50.7	47.6 ± 51.0	0.001
Oxigen delivery at admission:			
Low flow (Nasal cannulas/masks)	30/69 (43.5%)	172/318 (54%)	NS
High flow (C-PAP/HFNC)	40/69 (57.9%)	152/318 (47.8%)	NS
Mechanical ventilation	2/69 (2.9%)	70/318 (22%)	0.001
Medical therapy at admission:			
ACEIs, *n*	7/67 (10.5%)	40/318 (12.6%)	NS
ARBs, *n*	6/68 (8.9%)	34/318 (10.7%)	NS

**Table 7:** Exitus in COVID-19 positive patients: univariate analysis.

Patients, *n*	Exitus (*n* = 69)	No Exitus (*n* = 317)	*p*
Biochemistries at admission:			
IL-6, pg/mL	116.2 ± 95	89.7 ± 130.5	NS
LDH, IU/L	372.8 ± 151	337.8 ± 180.5	NS
PCR, mg/dL	12.4 ± 9.1	11.7 ± 23.6	NS
D-dimer, mcg/mL	2912.3 ± 4434	4624.3 ± 23778.6	NS
Ferritin, ng/mL	1831.5 ± 4337	1412.7 ± 1732.3	0.02
ALT, IU/L	152.5 ± 521.06	50.8 ± 60.2	0.001
White blood cells	9432.1 ± 4802	7985.5 ± 4518	0.04
AKI, *n*	39 (56.5%)	80 (25.1%)	0.0001
AKI stage 1, *n*	24 (34.7%)	50 (15.7%)	
AKI stage 2, *n*	10 (14.4%)	16 (5%)	
AKI stage 3, *n*	5 (7%)	13 (16.2%)	

**Table 8:** Exitus in COVID-19 positive patients: univariate analysis (in-hospital stay).

Patients, *n*	Exitus (*n* = 69)	No Exitus (*n* = 317)	*p*
Medical therapy during hospital stay:			
Antibiotics, *n*	64/69 (92.7%)	255/317 (80.4%)	0.01
Diuretics, *n*	37/69 (53.6%)	96/317 (30.3%)	0.001
FANS, *n*	17/69 (24.6%)	70/318 (22%)	NS
Glucocorticoids, *n*	26/69 (37.7%)	133/316 (42%)	NS
Heparin, *n*	54/69 (78.3%)	272/317 (85.8%)	NS
Hydroxychloroquine, *n*	57/69 (82.6%)	285/316 (90.2%)	NS
ACEIs/ARBs, *n*	23/69 (33.3%)	117/317 (36.9%)	NS
Antivirals, *n*	22/69 (31.9%)	125/317 (39.4%)	NS
Contrast medium, *n*	7/69 (10.1%)	65/318 (20.4%)	NS
Clinical parameters during hospital stay:			
MOF, *n*	3/69 (4.3%)	7/318 (2.2%)	NS
Sepsis, *n*	14/69 (20.3%)	53/318 (16.7%)	NS
COVID-19 related pneumonia, *n*	68/69 (98.5%)	304/318 (95.6%)	NS
HD, *n*	0	6/318 (1.9%)	NS
Severe COVID-19, *n*	49/51 (96%)	186/213 (87.3%)	0.04
Biochemistries during hospital stay:			
Serum creatinine, mg/dL	1.36 ± 0.88	0.9 ± 0.34	0.0001
Azotemia, mg/dL	84.09 ± 45.7	37.7 ± 30.4	0.0001
D-dimer, mcg/mL	6086.5 ± 10,709.0	1180.8 ± 1254.8	0.0001
IL-6, pg/mL	51.4 ± 65.1	38.2 ± 168.8	NS
ALT, IU/L	45.9 ± 51.3	52.2 ± 42.4	NS
LDH, IU/L	418.4 ± 179.8	206.2 ± 69.6	0.001
PCR, mg/dL	52.7 ± 317.2	1.26 ± 2.32	0.004
White blood cells	10,021.7 ± 5082.5	6736.7 ± 2364.6	0.0001

**Table 9:** Multivariable logistic regression to identify risk factors for mortality in COVID-19 positive patients (*n* = 284 pts).

	B	SE	Wald Test	*p*	Exp (B)
Age	0.109	0.027	15.969	0.000	1.115
Arterial hypertension	0.119	0.574	0.043	0.836	1.112
Cardiomiopathy	1.605	0.530	9.19	0.002	4.98
COPD	0.269	0.770	0.122	0.726	1.3
ACEIs/ARBs	−0.865	0.604	2.051	0.152	0.421
Creatinine	−0.11	0.419	0.069	0.792	0.85
Azotemia	−0.001	0.006	0.042	0.838	0.994
Ferritin	0.002	0.000	22.898	0.000	1.002
Chronic kidney insufficiency	0.386	0.894	0.186	0.666	1.471
Constant	−12.279	2.356	27.156	0.000	0.000

**Table 10:** Multivariable logistic regression to identify risk factors for mortality in COVID-19 positive patients (*n* = 165 pts).

	B	SE	Wald Test	*p*	Exp (B)
AKI stage (1)	1.446	0.625	5.363	0.021	4.248
AKI stage (2)	2.052	0.787	6.792	0.009	7.781
AKI stage (3)	−0.117	1.261	0.009	0.926	0.889
Ferritin	0.000	0.000	1.236	0.266	1.000
D-dimer	0.000	0.000	0.096	0.754	1.000
GB	0.000	0.000	11.610	0.001	1.000
Constant	−4.134	0.740	31.219	0.000	0.016

**Table 11:** Risk for in-hospital death among patients with SARS-CoV-2 infection and AKI stage 1 model 1 = adjusted for ferritin, D-dimer; model 2 = adjusted for ferritin, D-dimer, white blood cells; model 3 = adjusted for ferritin, white blood cells. Demographic and clinical parameters that resulted not significant at univariate analysis were not enrolled in multivariable models.

	HR (95% CI) or Wald Test (SE)	*p*
Unadjusted	3.85 (2.24; 6.60)	*p* < 0.001
Adjusted (model 1)	10.089 (±0.576)	*p* = 0.001
Adjusted (model 2)	5.363 (±0.625)	*p* = 0.021
Adjusted (model 3)	3.959 (±0.569)	*p* = 0.041

## Data Availability

Not applicable.

## Supplementary Materials

The following supporting information can be downloaded at: https://www.mdpi.com/article/10.3390/pathogens11111272/s1, Table S1: Characteristics of severe COVID-19 positive study patients (hospital admission); Table S2: Characteristics of severe COVID-19 positive study patients (admission and in-hospital stay).

## Abbreviations

ACEIs	Angiotensin Converting Enzymes
AE	Adverse Events
AH	Arterial hypertension
AKI	Acute kidney injury
ARBs	Angiotensin receptor blockers
CI	Confidence intervals
CKD	Chronic kidney disease
COVID-19	Coronavirus disease 2019
COPD	Chronic obstructive pulmonary disease
CPAP	Continuous positive airway pressure
DM	Diabetes mellitus
eGFR	Estimated glomerular filtration rate
ESRD	End-stage renal disease
FANS	Non-steroidal anti-inflammatory drugs
HD	Haemodialysis
HFNC	High flow nasal cannula
ICU	Intensive care unit
KDIGO	Kidney Disease: Improving Global Outcomes
NA	Not available
PRES	Posterior reversible encephalopathy syndrome
RRT	Renal replacement therapy
SARS-CoV-2	Severe acute respiratory syndrome coronavirus 2
UTI	Urinary tract infection
VM	Venturi mask
WHO	World Health Organization
