# Supplementary material for: Acute Kidney Injury in Non-Intensive Care Unit (ICU) Hospitalizations for Coronavirus Disease (COVID-19)
Source: Pathogens. 2022 Oct 31;11(11):1272. doi: 10.3390/pathogens11111272 (PMC9693191; doi:10.3390/pathogens11111272)
Supplement: Supplementary file 1 [file pathogens-11-01272-s001.zip › pathogens-1902422-supplementary.pdf]

Supplementary Table S1. Characteristics of severe COVID-19 positive study patients (hospital admission).

**Patients, *n***	***n* = 387**
Age, years	66.0±15.81
Males, *n*	247/387 (63.8%)
Positive medical history:	
-Arterial hypertension, *n*	174/387 (44.9%)
-Cardiomiopathy, *n*	98/387 (25.3%)
-Chronic kidney insufficiency, *n*	40/387 (10.3%)
-COPD, *n*	38/387 (9.8%)
-Diabetes mellitus, *n*	65/387 (16.8%)
-Malignancy, *n*	53/387 (13.7%)
-Peripheral arterial disease, *n*	50/387 (12.9%)
Biochemistry at admission:	
-Serum creatinine, mg/dL	1.59±7.34
-Azotemia, mg/dL	51.9±44
-PCR, mg/L	11.85±21.9
-IL-6, pg/mL	91.9±127.9
-D-dimer, mcg/mL	4420.1±22,368
-White blood cells, *n*	8234.3±4594.8
-Lymphocytes, *n*	1250.9±2154.9
-ALT, IU/L	67.0±216.7
-LDH, IU/L	342.3±177.3
-Ferritin, ng/mL	876 (26,714; 69)
Physical parameters at admission:	
-Blood pressure, mmHg	131.1±19.1/ 75.8±12.0
-Heart rate, bpm	88.4±17.3
-Body temperature, degree	37.9±7.8
-PaO_2/ FiO_2	267.12±102.5
-(Stage 0) PaO_2/ FiO_2 (>300)	153 (46.7%)/328
-(Stage 1) PaO_2/ FiO_2 (300-201)	89 (27.1%)/ 328
-(Stage 2) PaO_2/ FiO_2 (200-101)	60 (18.3%)/ 328
-(Stage 3) PaO_2/ FiO_2 ($\leq$100)	26 (7.9%)/ 328
Medical therapy at admission:	
-ACEIs, *n*	47/386 (12.2%)
-ARBs, *n*	39/386 (10.1%)

Supplementary Table S2. Characteristics of severe COVID-19 positive study patients (admission and in-hospital stay).

**Patients, *n***	***n* = 235**
AKI	86/235 (36.6%)
-AKI stage 1	56/ 86 (65.1%)
-AKI stage 2	17/ 86 (19.7%)
-AKI stage 3	13/ 86 (15.1%)
Respiratory support:	
-Low flow oxygen delivery, *n*	74/235 (31.5%)
-High flow oxygen delivery, *n*	182/235 (77.4%)
-Mechanical ventilation, *n*	71/235 (30.2%)
Clinical manifestations (and adverse events) during hospital stay:	
-SARS-CoV-2 -related pneumonia, *n*	372/387 (96.1%)
-Anaemia, *n*	8/235 (3,4%)
-Atrial fibrillation, *n*	7/ 235 (2.9%)
-Haemodialysis, *n*	5/ 235 (2.1%)
-Ischemic stroke, *n*	1/ 235 (0.05%)
-Multi-organ failure, *n*	7/ 235 (3.0%)

-PRES, *n*	3/235 (1.2%)
-Thrombosis, *n*	33/235 (14%)
-UTI, *n*	7/235 (2.9%)
-Others	28/235 (20.7%)
Concurrent medical therapy:	
-ACEIs and/or ARBs, *n*	95/235 (40.4%)
-Antibiotics, *n*	210/235 (89.4%)
-Antivirals, *n*	114/235 (48.5%)
-Diuretics, *n*	106/235 (45.1%)
-FANS, *n*	59/235 (25.1%)
-Glucocorticoids, *n*	125/235 (53.2%)
-Heparin, *n*	210/235 (89.4%)
-Hydroxychloroquine, *n*	217/235 (92.3%)
